# The Inborn Errors of Immunity—Virtual Consultation System Platform in Service for the Italian Primary Immunodeficiency Network: Results from the Validation Phase

**DOI:** 10.1007/s10875-023-01644-y

**Published:** 2024-01-17

**Authors:** Emma Coppola, Mayla Sgrulletti, Manuela Cortesi, Roberta Romano, Emilia Cirillo, Giuliana Giardino, Laura Dotta, Caterina Cancrini, Dario Bruzzese, Raffaele Badolato, Viviana Moschese, Claudio Pignata, in collaboration with IEI-VCS Task Force, Aiuti Alessandro, Donato Amodio, Chiara Azzari, Clementina Canessa, Roberta Caorsi, Riccardo Castagnoli, Maria Pia Cicalese, Francesca Conti, Rita Consolini, Rosa Maria Dellepiane, Andrea Finocchi, Vera Gallo, Marco Gattorno, Simona Graziani, Francesca Lippi, Vassilios Lougaris, Baldassarre Martire, Andrea Matucci, Antonio Marzollo, Cinzia Milito, Davide Montin, Giorgio Ottaviano, Paolo Palma, Giuseppe Patuzzo, Andrea Pession, Maria Cristina Pietrogrande, Alessandro Plebani, Isabella Quinti, Silvia Ricci, Francesco Saettini, Annarosa Soresina, Giuseppe Spadaro, Alberto Tommasini, Nino Trizzino, Stefano Volpi, Alessandra Vultaggio, Fiammetta Zunica

**Affiliations:** 1 Section of Pediatrics, Department of Translational Medical Sciences, Federico II University, Via S. Pansini, 5-80131 Naples, Italy; 2 Pediatric Immunopathology and Allergology Unit, Policlinico Tor Vergata, University of Tor Vergata, Rome, Italy; 3 PhD Program in Immunology, Molecular Medicine and Applied Biotechnology, University of Rome Tor Vergata, Rome, Italy; 4 Pediatrics Clinic and Institute for Molecular Medicine A. Nocivelli, Department of Clinical and Experimental Sciences, ASST- Spedali Civili of Brescia, University of Brescia, Brescia, Italy; 5 Research Unit of Primary Immunodeficiency, IRCCS Bambin Gesù Children Hospital, Rome, Italy; 6 Department of Public Health, Federico II University, Naples, Italy

**Keywords:** Inborn errors of immunity, Virtual Consultation System, IPINet, web-based platform, complex IEIs, undiagnosed IEIs

## Abstract

**Purpose:**

Inborn errors of immunity (IEI) represent a heterogeneous group of rare genetically determined diseases. In some cases, patients present with complex or atypical phenotypes, not fulfilling the accepted diagnostic criteria for IEI and, thus, at high risk of misdiagnosis or diagnostic delay. This study aimed to validate a platform that, through the opinion of immunologist experts, improves the diagnostic process and the level of care of patients with atypical/complex IEI.

**Methods:**

Here, we describe the functioning of the IEI-Virtual Consultation System (VCS), an innovative platform created by the Italian Immunodeficiency Network (IPINet).

**Results:**

In the validation phase, from January 2020 to June 2021, 68 cases were entered on the IEI-VCS platform. A final diagnosis was achieved in 35/68 cases (51%, 95% CI 38.7 to 64.2). In 22 out of 35 solved cases, the diagnosis was confirmed by genetic analysis. In 3/35 cases, a diagnosis of secondary immunodeficiency was made. In the remaining 10 cases, an unequivocal clinical and immunological diagnosis was obtained, even though not substantiated by genetic analysis.

**Conclusion:**

From our preliminary study, the VCS represents an innovative and useful system to improve the diagnostic process of patients with complex unsolved IEI disorders, with benefits both in terms of reduction of time of diagnosis and access to the required therapies. These results may help the functioning of other international platforms for the management of complex cases.

**Supplementary Information:**

The online version contains supplementary material available at 10.1007/s10875-023-01644-y.

## Introduction

Inborn errors of immunity (IEI) represent a heterogeneous group of rare genetically determined diseases, caused by a quantitative and/or functional defect in one or more immune system components [1, 2]. In the last two decades, novel diagnostic techniques, including newborn screening, have become available as an effective tool to achieve an early diagnosis and treatment. Moreover, the high throughput sequencing (HTS) technology has expanded our understanding of the genetic background of IEI [3–6]. To date, at least 480 monogenic forms of IEI have been identified, of which 65 identified in the last 2 years, and this number is growing year by year, thanks to the refinement of innovative technologies [2, 7]. Susceptibility to recurrent and/or severe infectious diseases has long been recognized as the clinical hallmark of IEI. However, over the last two decades, a clinical phenotype dominated by immune dysregulation (i.e., autoimmunity/hyperinflammation, malignancy, granulomatous and/or lymphoproliferative disease, enteropathy, and severe forms of atopy) has been increasingly recognized, even as the sole initial manifestation [8–16].

Due to the rarity, heterogeneity, and complexity of IEIs, which often lack clear hallmarks, they might represent a real challenge for clinicians, leading to a delay in the diagnostic process [17, 18]. On the other hand, an early diagnosis of IEI is crucial to prevent complications and organ damage. Recent advances in molecular and functional characterization of novel IEIs may provide a better understanding of their immunobiology and, whenever possible, may expand conventional treatment to a targeted or semi-targeted approach for the optimal management of these patients.

The European Society for Immunodeficiencies (ESID) Registry Working Party provides a continuous update of IEI Clinical Diagnostic Criteria [19] (https://esid.org/Working-Parties/Registry-Working-Party/Diagnosis-criteria). However, some patients with persistent immunological abnormalities do not fulfill the ESID diagnostic criteria for any defined clinical IEI. These cases represent a real challenge for the clinicians. In such cases, the opportunity of consulting multiple experts in the field may be helpful to obtain support that may improve the process of diagnosis and management of the patient. This approach favors an appropriate medical care and may reduce the suffering odyssey for patients and their families [20].

Actually, there are no published data on the diagnostic delay of IEI in Italy. A preliminary evaluation of IPINet data shows that 50% of patients receive the diagnosis within a year of the onset of symptoms. For the remaining 50%, the delay in diagnosis varies in a range from 2 to 10 years depending on the specific disorder. However, for patients with complex phenotypes, it is likely that the latency between the onset of symptoms and diagnosis is even longer.

Thus, to meet the need for early identification of complex, atypical, yet undiagnosed IEI disorders, the IEI-Virtual Consultation System (VCS) program has been implemented and developed within IPINet. This digital platform consists of an innovative tool for remote clinical consultation that allows a joint evaluation of complex clinical cases by IPINet experts, to offer a web-based second opinion system.

Hereby, we describe the infrastructure and functioning of IEI-VCS and report the results of the validation phase.

## Methods

### IEI-VCS Platform

The IEI-VCS aimed at improving the diagnostic process and the management of IEI complex clinical cases, not fulfilling ESID diagnostic criteria, by remote sharing and multidisciplinary digital consultation among IPINet experts. Briefly, IEI-VCS platform primarily aimed to (I) reach a consensus opinion among IPINet immunology experts regarding complex/atypical IEI cases, (II) reach a diagnosis and improve the level of care for patients with undiagnosed diseases, (III) improve/revise diagnostic and therapeutic management protocols for complex and rare IEI cases, and (IV) identify putative pathogenic mechanisms of new and rare diseases, in the perspective of a precision-medicine approach. Secondary endpoints included the following: (I) the development of a valuable database on atypical and**/**or complex IEI cases, as an Undiagnosed Board-Repository to facilitate collaborative research, (II) the evaluation of the clinical outcomes following multidisciplinary discussion.

### Ethical Compliance

The IEI-VCS platform has been created by the not-for-profit Consortium *CINECA* that is made up of 67 Italian Universities, 9 Italian Research Institutions, 1 Polyclinic, and the Italian Ministry of Education. CINECA infrastructure is certified for quality and security procedures: the *ISO 9001:2008 quality certification* for “Design, development, creation, and distribution of services and systems in the field of Information and Communication Technology”, and *the ISO 21001:2013 security certification* for “Analysis, design, development, operation and maintenance of Decision Support Systems and Information Systems Infrastructures for management, monitor, and analysis of clinical trials and epidemiological registries for health services organizations”. CINECA is compliant with Computer System Validation guidelines and owns a service named *Conserva* for digital preservation. In addition, CINECA provides identification and access tracking with Audit Trail (compliant with Italian SPID), that allows accounting data to be traced to its source, a disaster recovery system, that enables the resumption of data, and that guarantees the anonymization and pseudonymization of personal data. The access is restricted to Centers that have been accredited by IPINet. The service allows the user to run the access to the platform anytime, anywhere from any web-enabled devices (Windows, Mac), including mobile devices and tablets; no installation is needed. Through HTTP and SSL protocols and thanks to the access limited via username and password, high levels of security and confidentiality of information are guaranteed. The platform offers a secure, encrypted data transfer channel that protects all sensitive information in transit and at rest. The service provides comply with the new European regulation on the protection of privacy (n. 679/2016). The system is aimed to the IT integration with the most widespread protocols and standards used in the field of authentication and authorization (LDAP, CAS, Shibboleth, etc.). It is also possible to federate the authentication system with other authentication systems that support the SAMLv2 standard. File transfer is available from Electronic Health Records or other Healthcare Systems (HL7 and IHE health interoperability protocols). Data exchange is allowed in the most common formats (Excel, etc.), throughout automatic data loading procedures. All data were collected and shared in anonymized form, in accordance with GDPR and/or local privacy policies. In respect of anonymity, the patient is identified only by the initials; the only sensitive data reported in the system is the date of birth. Informed consent for data collection and sharing in the IEI-VCS platform has been obtained in written form from each patient or his/her legal guardians by the treating physician. A local ethical committee approved the study.

### Patients’ Selection and Data Collection

Within the IEI-VCS project, all IPINet Centers shared the following characteristics as follows: (I) University Centers or III level Hospitals committed to IEI care, (II) Units exclusively dedicated to IEI care, (III) Centers recognized at the National and International level with expertise in the field of IEI, either in the clinical management of patients and in related scientific activities. Project Coordinating Centers are the following: Federico II University of Naples (Prof. Claudio Pignata), University of Rome Tor Vergata (Prof. Viviana Moschese), and University of Brescia (Prof. Raffaele Badolato). In this validation phase, clinical cases were enrolled by the following IPINet Centers: Pediatric Immunology Center, Federico II University, Naples; Pediatric Immunopathology and Allergology Unit/Regional Referral Center for PIDs, Tor Vergata University Hospital, Rome; Infectious Diseases Division, Bambino Gesù Children’s Hospital, Rome; Department of Clinical and Experimental Sciences, University of Brescia. The study population included both pediatric and adult patients with an atypical or complex clinical-immunological phenotype, who did not fulfill ESID diagnostic clinical criteria. Data were prospectively collected for each patient in an electronic case report form (eCRF). Information on patients’ demographic data, family and personal clinical history along with immunological laboratory finding, and genetic characterization, routinely performed in accordance with current guidelines and/or local standard of care, as well as details on past and current treatments, have been included in eCRF. In order to provide IPINET Clinicians with all information required for the most appropriate diagnostic and therapeutic indications, DICOM-type images (e.g. MRI, CT, PET, PET-CT, SPECT-CT, ultrasound) and pathology images (e.g. slides) could be uploaded into the IEI-VCS platform, when required. The system also includes a web viewer for diagnostic investigations with the potential for magnification, color contrast processing, and distance measurement.

The inclusion criteria were as follows:

- IEI patients, with clinical and laboratory features who do not fulfill ESID clinical diagnostic criteria, but
  - with persistent documented immunological abnormalities with/out family history highly suggestive of IEI,
    or
  - associated to complex and atypical extra-immunological signs,
    or
  - with negative genetic investigation for the most suggestive genes consistent with that specific phenotype;

- Patient’s or legal guardian’s agreement to participate to the IEI-VCS consultation system;
- Patient’s ability and willingness to be engaged in additional clinical and research workup.

Before enrolling the case, IPINet Clinicians were actively required to (a) obtain consent from the patient or his/her legal guardians for IEI-VCS teleconsulting medical advice; (b) acknowledge that opinion does not constitute a formal consultation; and (c) declare that the treating clinician bears legal medical responsibility for the patient. Disclaimers have been consulted and approved by European and US lawyers.

eCRF collected data included clinical (family history, personal history of recurrent/atypical/severe infections, immune dysregulation, syndromic features) and immunological work-up, in line with best clinical practice (complete blood count, complement evaluation, Ig and IgG subclasses levels, standard and extended T and B immunophenotype, autoimmunity screening tests and any functional tests carried out according to patient clinical phenotype), imaging and, whenever available, genetic analysis. Moreover, data on past and current treatments were collected for each patient.

### IEI-VCS “Immunology Experts” and Platform Workflow Functioning

**Fig. 1:**
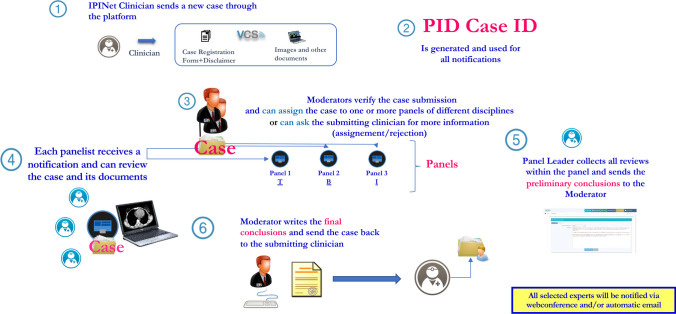
Workflow for IEI-VCS. Clinicians enroll the case and the coordination team approves the case enrollment once the eligibility criteria are evaluated. The coordination team assigns the case to one or more competent panel(s), according to the prevalent immune disturbance. The panel leader assigns the case to one or more panel members. The panelist examines the case and comments on diagnoses, therapeutic interventions, and/or suggestions for further investigations. The panel leader collects all the panelists’ reviews and draws up the preliminary conclusions. Then, coordinators have the key role of editing the final conclusions, which are sent back to the referring clinician

Figure 1 illustrates platform workflow. Several IPINet immunological experts (panelists), both senior and junior, were grouped into three Panels: innate immunity (Panel I), cellular immunity (Panel T), and humoral immunity (Panel B). A referral expert (Panel Leader) was identified for each panel, with the task of supervising and coordinating the panelist’s activities. The role of each IEI-VCS expert is detailed in Table 1. The IPINet Clinician enrolls the case and the coordination team (CoT) approves the enrollment according to eligibility criteria. Further, CoT keeps tracking of diligent process progression up to completion. Each single IEI-VCS expert has a distinct role according to the consultation’s different steps as per a hierarchical organization. Any IEI-VCS expert can monitor at any time the status of enrollment, date of request, and workflow consultation profile, up to final review. CoT assigns the case to one or more expert Panel(s), according to the prevalent immune defect (innate, cellular, humoral defects). The distribution of Italian IPINet Centers is shown in the Supplementary Figure.

**Table 1:** IEI-VCS immunology performers’ roles

*The Clinician of an IPINet Center*	• Sends a new case through the platform; • Before proceeding with the case registration, must confirm the disclaimers, compile the English e-CRF of the registered case, fulfilling the requested data, and upload the related supporting documents, including diagnostic images and congruent data. All personal DICOM standard metadata associated to the patient are anonymized in the process of upload. Any optional non-DICOM metadata upload requires previous anonymization by the referring clinician; • Following the consultation, the referring clinician must fill in the VCS questionnaire to explain if and how the advice has been useful for the management of the IEI patient, and to make any further observations
*The Coordinating Team (3 Members)*	• Is responsible for the approval of the case (approximately by 15 days); • Check the case for completeness and can ask the submitting clinician to add further information, if necessary; • Forwards the case to one or more competent panel leader/s; • Must ensure a timely review/closure of each case, validate the consensus, and summarize recommendations which will be sent to the referring clinician (approximately by 30 days)
*The Panel Leader*	• Has the role of assigning the case report to one or more panelists and of facilitating the discussion among them; • When required, can schedule web meetings for discussion, that is registered in the calendar section of the web page and whose notification is sent to any panelist by email; • Collects all reviews within the panel to provide the consensus and validates the preliminary conclusions to be sent back to the Coordinating Team on time
*The Panelists*	• Are senior or junior experts in the field of inborn errors of immunity, grouped in three distinct Panels (T lymphocyte defects, B lymphocyte defects, Innate immunity defects); • Are involved in the case evaluation, according to the prevalent immune disturbance; • Interact with the panel leader; • Receive the notification of a newly submitted case via an automatic e-mail message; • Can review the case and its documents and complete the review e-CRF by the time due

### Expert Review

One or more Panel Leader(s) launches the case to the corresponding panelists. The active panelist examines the case on the platform and fills out specific items regarding diagnosis, therapeutic interventions, and/or suggestions for further investigations. A blind activity is performed by each panelist. Upon collection of panelists’ revisions, the Panel Leader writes the report and the CoT finalizes it to be sent back to the IPINet referring clinician. In case of panel disagreement, the CoT may open an online discussion forum with all experts before posting the final report.

IEI-VCS platform is integrated with the Human Phenotype Ontology (HPO) System (https://hpo.jax.org). Each patient’s clinical data is associated with a corresponding HPO code, allowing the generation of a “Disease Report” and “Genes Report” linked to the specific IEI-VCS case. All IEI-VCS immunology experts, throughout all the revision steps, were able to benefit from these reports.

### Data Quality Assurance

Data quality is under the responsibility of each individual IPINet expert. The coordinating team is required to check data completeness for each eCRF filled by the IPINet Clinician and, when required, may ask the clinician to supplement the patient’s information.

### Effectiveness

To evaluate the effectiveness of IEI-VCS in the diagnosis of complex cases to support the clinicians, the following items were evaluated:

- The percentage of cases where at least one diagnostic hypothesis was provided by the panels;
- The percentage of agreement between the suggested diagnosis and the definitive diagnosis after panel revisions;
- The interrater panelist agreement within the same panel. The agreement was expected to be lower in the case of less defined phenotypes and with more extra-immunological features or in cases where the clinical-immunological phenotype was not supported by informative genetics;
- The interrater agreement among different panels.

### Statistical Analysis

All statistical analyses were performed using the R platform (vers. 3.9 or next) R Core Team (2021). R: A language and environment for statistical computing. R Foundation for Statistical Computing, Vienna, Austria (https://www.R-project.org/).

Percentages and proportions are reported for demographic variables and clinical and immunological features. Interrater panelist/panels agreement and agreement between the suggested diagnosis and the definitive diagnosis were assessed as a percentage, and the 95% confidence interval was calculated.

## Results

### Data Entered in the Platform

#### Patients

**Fig. 2:**
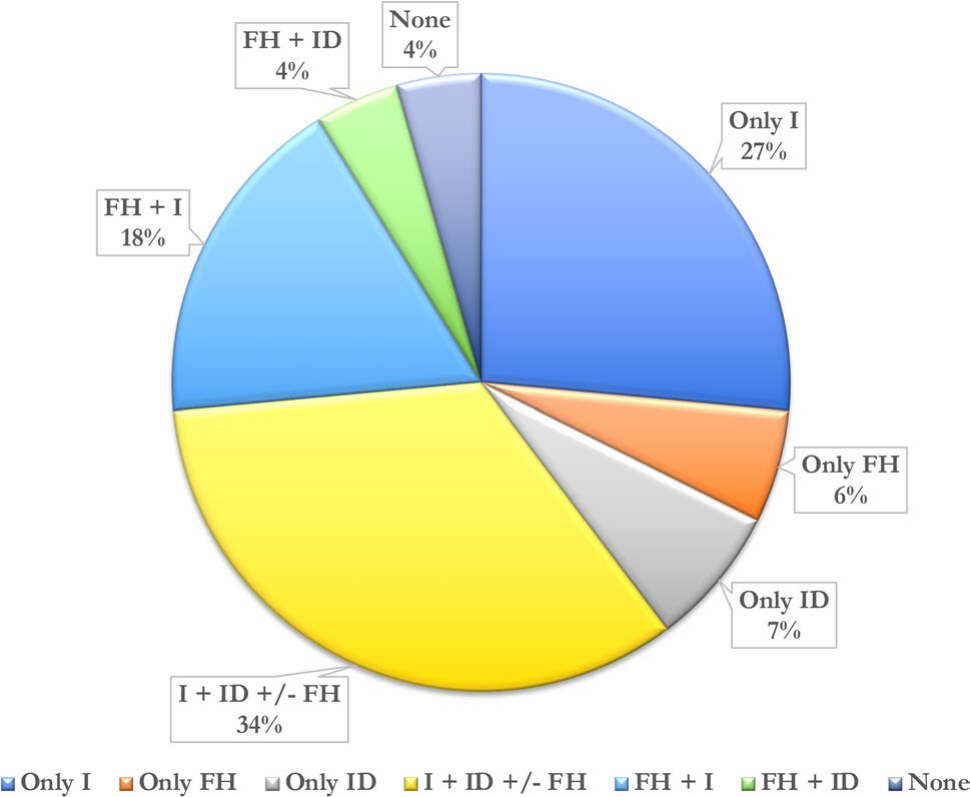
Distribution and association of the main clinical characteristics of the cohort. Legend: FH family history, I infections, ID immune dysregulation

From January 2020 to June 2021, 68 cases were entered on the IEI-VCS platform. The main features of the patients are summarized in Table 2. Patients’ age range was between 3 and 66 years (median age 17 years). Twenty-two were adult patients. Forty-three were males. Fifty-nine patients were of Caucasian origin, 5 Asian, and 3 Black–African. Sixty-five patients (95%) had at least one of the following: (a) positive family history for IEI (38%); (b) history of infections (53%); (c) manifestations of immunodysregulation (71%), as shown in Fig. 2. Four percent (3/68) exhibited only immunological abnormalities in the absence of a clinical related phenotype: one patient had hypogammaglobulinemia and decreased CD19 + and CD4 + cells, second had decreased TH17 and third hypogammaglobulinemia and decreased CD19 + cells. The most frequent immunological alterations were hypogammaglobulinemia (39%), low number of CD4 + cells (33%), low number of CD19 + cells (30%), lymphopenia (23%), serum IgE levels > 2000 UI/L (19%), neutropenia (11%), and low number of TH17 + cells (8%). Thirty percent of patients had only one laboratory abnormality, and 51% had two or more.

**Table 2:** Clinical and laboratory features of the cohort

Variable	Cohort (*N* = 68)
Age-yr Median age	3–66 17
Sex—no. (%)	
Male Female	43 (63) 25 (36)
Race or ethnic group—no. (%)	
White/Caucasian Asian Black/African Other	59 (86) 5 (7) 3 (4) 1 (1)
Family history—no. (%) For defined IEI For autoimmunity Other	26 (38) 12 (17) 13 (19) 15 (22)
Asymptomatic patients with positive family history—no. (%) Asymptomatic patients without positive family history—no. (%)	4 (6) 3 (4)
Infections—no. (%) Recurrent respiratory infections Abscesses—skin infections Urinary tract infections GI infections Candidiasis CNS infections Sepsis Severe infections	53 (77) 37 (54) 22 (32) 8 (12) 8 (12) 7 (10) 5 (7) 3 (4) 39 (57)
Patients with only history of infections	18 (27)
Autoimmunity—no. (%) Type 1 diabetes mellitus Coeliac disease Autoimmune thyroiditis Enteropathy—IBD ITP	16 (23) 2 (3) 4 (6) 2 (3) 2 (3) 5 (7)
Allergy—no. (%) Atopic dermatitis Food allergy Asthma Rhinoconjunctivitis	13 (19) 12 (17) 4 (6) 3 (4) 2 (3)
Malignancies—no. (%) Solid tumor Hematological tumor	4 (6) 1 (1.5) 3 (4)
Lymphoproliferation—no. (%) Hepatomegaly Splenomegaly Lymphadenopathy GLILD	16 (23) 9 (13) 8 (12) 6 (9) 1 (1.5)
Patients with only immune dysregulation	5 (7)
Others—no. (%)	5 (7)
Laboratory abnormalities—no. (%) Anemia Leukopenia Thrombocytopenia Neutropenia Lymphopenia Hypogammaglobulinemia Hypergammaglobulinemia Hyper-IgA Hyper IgM Hyper IgE Low specific antibody response (PCP) Low CD4^+^ Low CD8^+^ Low CD3^+^ Low CD19^+^ Low CD16 Low TH17 Low-switched memory B cells Low T Reg High CD21 low B cells Autoantibodies positivity Low complement Others	61 (90) 18 (26) 6 (9) 5 (7) 8 (11) 16 (23) 27 (39) 5 (7) 5 (7) 2 (3) 13 (19) 11 (16) 23 (33) 15 (22) 11 (16) 21 (30) 6 (9) 6 (9) 1 (1.5) 1 (1.5) 1 (1.5) 14 (20) 1 (1.5) 43 (63)

### Panel Assignment

**Fig. 3:**
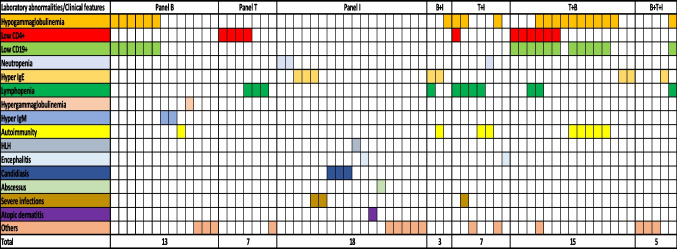
Assignment to panels based on the main clinical-immunological characteristics of the patient. In the figure, each column represents a single patient. The clinical and laboratory characteristics that guided the assignment to the reference panels are highlighted with different colors

According to the prominent immunological defect, the cases were assigned to one or more panels, as shown in Fig. 3. In particular, patients referred for humoral abnormalities were assigned to Panel B (13, 19%), patients with cellular abnormalities were assigned to Panel T (7, 10%), and patients with clinical and laboratory features suggestive of quantitative/qualitative phagocyte abnormalities or with an immunological phenotype suggestive of innate defects were assigned to Panel I (18, 26%). Thirty (44%) patients, with no univocal features, were assigned to two or more Panels.

#### Evaluation of the Panelist’s Activity

In general, at least one hypothesis was provided in 72% of cases. In all cases, further laboratory and/or instrumental were requested for an in-depth evaluation.

**Fig. 4:**
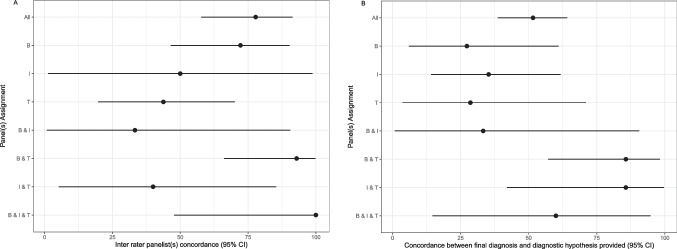
Evaluation of the activity of the panelists. **a** Concordance between the panelists on diagnostic hypotheses within the individual panel (B, I, T) and agreement between different panels (B and I, B and T, I and T, B and I and T). **b** Concordance between the final diagnosis and the diagnostic hypothesis provided by the panelists. Horizontal bars indicate 95% CI; closed dots indicate the percentage of concordance

The percentage of diagnostic agreement among panelists of the same panel was variable. In Fig. 4A, the diagnostic concordance of panelists within the same panel and among different panels is illustrated. Of the 36 cases assigned to Panel B, 18 were evaluated by two panelists: in 13/18 cases, the same diagnostic hypothesis was provided, with a good percentage of agreement equal to 72% (95% CI 46.52 to 90.31). Of the 34 cases assigned to Panel T, 16 were evaluated by two panelists: 7/16 cases received the same diagnostic hypothesis with a fair percentage of agreement of 43% (95% CI 19.75 to 70.12). The agreement among panelists of Panel I could not be evaluated since only 2 cases were evaluated by two different panelists. When the case was assigned to more than one Panel, the percentage of interrater diagnostic agreement was found to be high. Panels T and B evaluated 15/68 cases, and agreement was found in 13/15 of them (93%, 95% CI 66.13 to 99.82). Panels T and I were assigned 7/68 cases with a diagnostic concordance in 2/7 cases (40%, 95% CI 5.27 to 85.34). Panels I and B were assigned 3/68 cases, and in one case, the diagnostic hypothesis matched (33%, 95% CI 0.84 to 90.57). Five of the 68 cases were assigned to the 3 panels, and in all of them, a diagnostic agreement was achieved (95% CI 47.82 to 100).

### Concordance with Final Diagnosis

Upon complete evaluation of the cases, diagnostic finalization was reported to the referring IPINet clinician. In Fig. 4B, the agreement between the final diagnosis and the panelists’ diagnostic hypothesis is reported. The final diagnosis matched the diagnostic hypothesis in 35/68 cases (51%, 95% CI 38.7 to 64.2). Of note, the highest agreement was found when the case was assigned to more than one panel, as per a more complex disorder. In particular, the agreement was 85% in B and T and T and I assignments. In 22 out of 35 solved cases, the diagnosis was confirmed by genetic analysis (see Supplementary Table). In 3/35 cases, a diagnosis of secondary immunodeficiency was made. In 10 out of 35 cases, the diagnostic hypothesis was confirmed by final clinical and immunological diagnosis but not unequivocally ascertained by genetic analysis. In 6/10, either a heterozygous mutation of a gene strongly suggestive of that specific phenotype but with an autosomal recessive inheritance was identified or a variant of uncertain significance (VUS) in a candidate gene was revealed. Of the 33 unsolved cases, 10 did not receive a diagnostic hypothesis; and in 23 cases, the proposed diagnosis was not confirmed by the final diagnosis. Four of these patients were diagnosed with an extremely rare syndrome such as interstitial lung disease, nephrotic syndrome and epidermolysis bullosa (ILNEB) syndrome, mirage syndrome, prolidase deficiency, PLCG2-associated antibody deficiency, and immune dysregulation (PLAID) syndrome. In all these cases the diagnosis was confirmed by genetics.

## Discussion

To address underdiagnosed, misdiagnosed, and diagnostic delays of individuals with IEI, we have implemented and developed the IEI-VCS, which is an innovative digital platform for remote clinical consultation. The project is aimed at better defining patients who have a complex clinical-immunological phenotype, do not fulfill ESID diagnostic criteria, are at risk of diverse diagnostic trajectories with inappropriate utilization of healthcare services, and with a high risk of misdiagnosis [21, 22]. The main purpose of the project is to obtain a “consensus opinion” on multifaceted or atypical IEI cases to respond to the needs of the patients, the doctors in charge, and the community for optimal care. The SARS-CoV2 pandemic has significantly affected and expanded some aspects of patient management well beyond in-person visits with increasing interest for remote consultation through telemedicine [23]. Telemedicine has been proven fundamental in the patient suffering from chronic diseases, for the continuity of care and the management of exacerbations. Even patients with undiagnosed diseases were able to benefit from remote consultation, avoiding the stall of the diagnostic process and the lack of access to life-saving treatments [24, 25]. Recently, the CPMS has been launched at the European level to acquire multiple opinions on unsolved issues of IEI disorders, representing a tool to help manage these patients [26, 27]. In our study, the VCS digital platform for patients not fulfilling ESID diagnostic criteria has been validated, and the effectiveness was analyzed, in terms of concordance between the diagnostic hypothesis provided by the IEI experts and the final diagnosis. The concordance in the whole patient cohort was considerable, with a good percentage of 51% of the cases. In real-life experience, the management of the complex or atypical IEI phenotype is usually characterized by a long and difficult diagnostic process, which often lasts months or even years with a huge waste of resources in terms of inappropriate investigations, frequent hospital accesses, and inadequacy to obtain a timely, correct and, sometimes, life-saving, therapeutic plan. In the context of IEI-VCS, the experts, with the sole analysis of patient data obtained from the CRF, were able to guide the diagnostic process and, in most cases, to define one or more diagnostic hypotheses. Indeed, for all cases, regardless of the occurrence of a specific diagnostic hypothesis, the experts have suggested laboratory, instrumental, or genetic investigations helpful for the subsequent management and for further elucidation of the case. Reaching a diagnosis in 51% of undiagnosed cases represents a significant effect on the quality of care. From the patient’s family perspective, acquiring a second opinion on their child’s challenging case represents a benefit in terms of a better “patient journey” experience.

A potential limitation of this preliminary study, mainly aimed at validating the system, is the limited number of cases. It is noteworthy, however, that these patients are affected with very rare diseases. Furthermore, in the field of rare IEIs, those patients with complex atypical phenotypes, as such, are precious to highlight previously unknown biological aspects.

The analysis of the concordance between the diagnostic hypothesis of the panel(s) and the final diagnosis showed that the percentage of concordance increases to 85% when the case is addressed by the binomial T and I or T and B. Conversely, the percentage of concordance is lower if the case is assigned to a single panel or to the binomial B and I, pointing to the rather heterogeneous and less codified profile of these cases.

Interestingly, from our data emerged that the final diagnosis allowed to allocate novel clinical features to already known complex inherited rare genetic disorders, such as PLAID, mirage syndrome, ILNEB, or prolidase deficiency. In this perspective, IEI-VCS represents a useful tool to expand our knowledge on the phenotype of diverse rare complex disorders.

Although genetic analysis remains fundamental for better characterization and understanding of IEI disorders [28, 29], in a significant proportion of patients, molecular sequencing is elusive due to technological constraints. In 10 out of 35 cases, functional studies have captured the defect underlining the importance of combined clinical and functional data to move forward with a targeted treatment, regardless of a genetic confirmation. Recently, epigenetic alterations have emerged in their role in shaping the immune response and as novel pathogenic mechanisms of rare IEIs [30, 31]. The advent and optimization of more advanced OMICS technologies will improve the deciphering of these rare conditions in the very near future [32]. Nowadays, since the management of complex rare diseases is challenging, a multicomponent diagnostic approach is recommended to improve diagnostic yield and successful interventions and sustainability in previously unrecognized IEI diseases.

Overall, IEI-VCS represents a value-making tool for data sharing and the diagnostic-making process across IEI experts to ameliorate complex IEI diagnosis and care. The results of this study may be complementary to those of other international platforms aimed at solving complex cases. Following the launch of the platform, in the future, it will be possible to analyze its impact on therapeutic strategies definition and on the outcome of these patients.

### Supplementary Information

Below is the link to the electronic supplementary material.

Supplementary Figure The distribution of the centers across Italy. The blue color indicates centers with a maximum of two IPINet expert immunologists; with red the centers with 3 or more. (PDF 58 KB)

Supplementary Table (DOCX 27 KB)

## Data Availability

The data that supports the findings of this study are collected in the VCS platform created by CINECA. Access is reserved for Centers accredited by IPINet. Data can be shared by the authors upon request.

## Abbreviations

CPMS: Clinical Patient Management System
CT: Computer tomography
CoT: Coordination team
PET: Positron emission tomography
eCRF: Electronic case report form
DICOM: Digital Imaging and Communications in Medicine
HPO: Human phenotype ontology
HTS: High throughput sequencing
ERN: European Reference Network
ESID: European Society for Immunodeficiencies
GDPR: General data protection regulation
IEI: Inborn errors of immunity
ILNEB: Interstitial lung disease, nephrotic syndrome, and epidermolysis bullosa
PLAID: PLCG2-associated antibody deficiency and immune dysregulation
IPINeT: Italian Immunodeficiency Network
MRI: Magnetic resonance imaging
PID: Primary immunodeficiency
SARS CoV2: Severe acute respiratory syndrome coronavirus 2
VCS: Virtual Consultation System
VUS: Variant of uncertain significance
