# Supplementary figures and images for: The Inborn Errors of Immunity—Virtual Consultation System Platform in Service for the Italian Primary Immunodeficiency Network: Results from the Validation Phase
Source: J Clin Immunol. 2024 Jan 17;44(2):47. doi: 10.1007/s10875-023-01644-y (PMC10794402; doi:10.1007/s10875-023-01644-y)

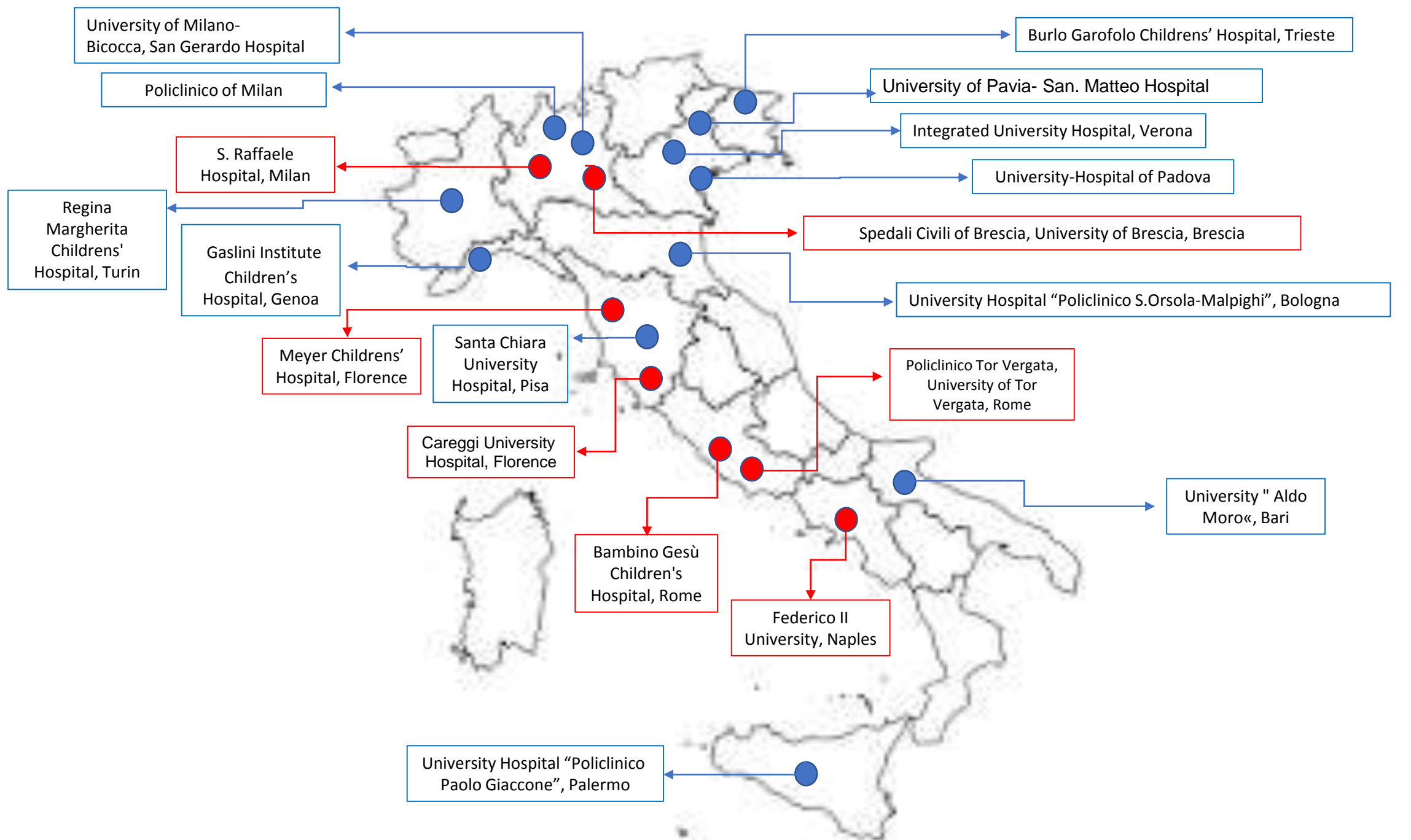

**Legend:** IPINet immunological experts    ●  $\leq 2$     ●  $> 3$

Supplement: Supplementary file 1 — Supplementary Figure The distribution of the centers across Italy. The blue color indicates centers with a maximum of two IPINet expert immunologists; with red the centers with 3 or more. (PDF 58 KB) [file 10875_2023_1644_MOESM1_ESM.pdf]
