# Supplementary material for: The Inborn Errors of Immunity—Virtual Consultation System Platform in Service for the Italian Primary Immunodeficiency Network: Results from the Validation Phase
Source: J Clin Immunol. 2024 Jan 17;44(2):47. doi: 10.1007/s10875-023-01644-y (PMC10794402; doi:10.1007/s10875-023-01644-y)
Supplement: Supplementary file 2 — Supplementary Table (DOCX 27 KB) [file 10875_2023_1644_MOESM2_ESM.docx]

**Supplementary Table. Supplementary Table. Genes identified in 22 cases with diagnosis confirmed by genetic analysis.**

| **Classification of inborn errors of immunity by International Union of Immunological Societies Expert Committee 2022.** | **Gene (n of patients)** |
| --- | --- |
| Hyper IgE Syndrome | STAT3 (1) |
| Defects in intrinsic and innate immunity | STAT1 (3)  TLR3 (1)  MYDD88 (1)  TICAM1 (1)  RANBP2 (1) |
| Bone marrow failure | SAMD9 (1) |
| Diseases of immune dysregulation | AP3B1 (1)  RANBP2 (1)  AIRE (1)  PEPD (1)  FOXP3 (1) |
| Predominantly antibody deficiencies | NFKB1 (2)  NFKB2 (1)  BAFFR (1)  BTK (1)  TACI (1) |
| Immunodeficiency affecting cellular and humoral immunity | DOCK8 (1) |
| Autoinflammatory disorder | PLCG2 (1) |
